# Psychological capital in university students: analysis of scientific activity in the scopus database

**DOI:** 10.1016/j.heliyon.2022.e11849

**Published:** 2022-11-25

**Authors:** Jesús Manuel Guerrero-Alcedo, Lorena C. Espina-Romero, Jessica Paola Palacios Garay, Freddy Roberpierre Jaimes Álvarez

**Affiliations:** a Universidad Científica del Sur, Lima, Peru; b Escuela de Postgrado (EPG-USIL), Universidad San Ignacio de Loyola, 15024 Lima, Peru; c Universidad Cesar Vallejo, Lima, Peru

**Keywords:** Psychological capital, Psycap, Students, University, Bibliometric review

## Abstract

Lately there has been an increase in the number of publications on psychological capital, especially in the specialty of organizational psychology, and not so much in the field of educational psychology. This study aims to analyze the main bibliometric indicators of production, collaboration and impact of scientific literature related to psychological capital in university students. We searched the Scopus database for documents using the comprehensive search strategy until June 27, 2021. Data were collected regarding title, keywords, authors, co-authors, citations received, details of the most productive journals, year of publication, countries, and institutions. From 2009 to 2021, 82 documents were published, mainly from countries such as China, Australia, the United Kingdom, the United States and Spain; the most productive journal was the so-called *Revista Argentina de Clínica Psicológica* while Curtin University was the most influential institution in the subject of study. The author's co-occurrence network analysis, trend topics, and keyword analysis highlighted themes involving psychological capital with educational and health variables in college students. The findings can help both academics and professionals to obtain an overview of advances in the subject and thus identify important gaps in the literature and propose promising new lines of research.

## 1 Introduction

In recent years there has been an increase in the number of publications on psychological capital (PsyCap) within the framework of positive psychology. Since its inception this construct has been used in the area that addresses positive organizational psychology (Abraham et al., 2020; Luthans, 2002; Melão and Reis, 2020); however, it has recently been extended to other areas such as positive psychology applied to education. In this area, psychological capital is defined as the study and application of human strengths and psychological capacities with positive orientation, which can be measured, developed, and managed effectively for the improvement of academic performance and results, which also leads to an increase in the general well-being of students and teachers (Martínez et al., 2019).

Following the approaches of Luthans et al. (2015), and applying their contributions to the educational field, it can be pointed out that psychological capital allows optimal development in individuals, in which it is possible to have confidence to make decisions and make the necessary effort to achieve success in academic tasks (self-efficacy), persevere until obtaining academic goals or redirect them to achieve them (hope), make positive attributions about present and future academic events (optimism) and recover from situations overwhelming or adverse academics and coming out strengthened (resilience) for the attainment of academic achievement (Martínez et al., 2021).

The scientific literature has shown that psychological capital is a good predictor of performance and satisfaction when evaluated globally and not so much when it is done for each specific dimension (Luthans et al., 2007). In addition, each of these resources are developed by the individual and may vary depending on circumstances and context, rather than being fixed, static personality traits (Dello Russo and Stoykova, 2015; Gülen Ertosun et al., 2015). It can also be evaluated in multiple domains of the person beyond the academic and work context such as health, relationships, and life itself in general (Luthans et al., 2013).

Within the university academic scenario, psychological capital has been directly related to variables such as academic performance, academic satisfaction and academic commitment (Sánchez-Cardona et al., 2021; Slåtten et al., 2021; Vîrgă et al., 2020), academic adjustment (Hazan Liran and Miller, 2019; Raza et al., 2020), orientation to learning objectives and academic satisfaction (Sánchez-Cardona et al., 2021), intrinsic motivation (Siu et al., 2014), academic exhaustion or boredom (Vîrgă et al., 2020), creativity (Hong et al., 2020), stress arising from academic expectations (Adil et al., 2020; Poots and Cassidy, 2020; Sifat, 2020), conscious learning and participation in learning (Lin, 2020) and comprehensive quality of higher education (Jin, 2020).

The studies have tried to explain the relationship between psychological capital and the variables associated with academic results. However, they have not examined bibliometric variables around the subject. Given this, the use of bibliometrics allows to identify quantitative variables that are essential for a certain area of scientific knowledge, since it tries to collect information on a particular topic, highlighting the relevant authors for the field of study, the number of publications, the keywords that will allow to establish the relationships with other variables of educational interest, collaborations of authors and data from journals, countries and research centers that stand out in terms of publications and their impact. Likewise, bibliometrics allows the implementation of scientific cartography techniques (for example, topic dendrogram, conceptual map and figures of trend topics, among others) as a tool to organize and analyze scientific information (Aria and Cuccurullo, 2017).

The evaluation of the scientific production around the psychological capital in university students will provide the identification of an overview of the international contribution in terms of this theme, the current issues discussed by the researchers and the research gaps in this field. Following the proposal of Zupic and Čater (2015) about bibliometric studies, the following research questions have been established: What is the global trend of scientific publications on psychological capital in university students? What information is discovered about this trend? What are the future lines of studies in this field?

This review aims to analyze the main bibliometric indicators of production, collaboration and impact of scientific literature related to psychological capital in university students. This includes the identification of authors, countries, journals, and institutions that are active and at the forefront of research related to the subject in question. It also aims to identify themes and trends in scientific production to inspire and generate directions for future studies.

## 2 Materials and methods

**Figure 1:**
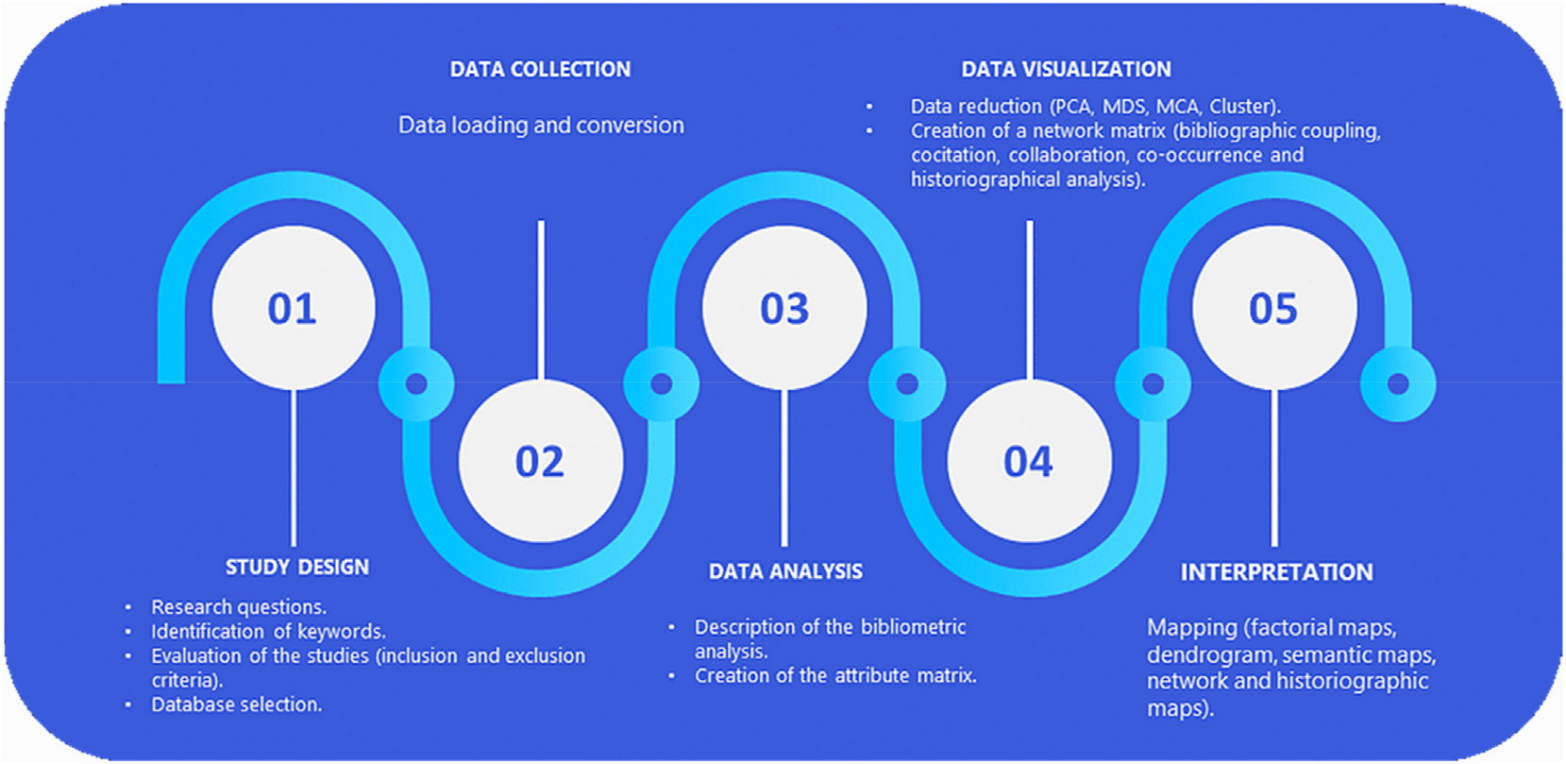
Methodological process for the realization of the bibliometric analysis.

The present study makes use of bibliometric mapping analysis. This method is recently being used in different scientific disciplines, gaining ground among academics who see its ideal use for conducting bibliometric reviews (Aria and Cuccurullo, 2017). Zupic and Čater (2015) have described five rigorous steps for its realization, which contemplate the design of the study (research questions, identification of keywords, inclusion and exclusion criteria, database selection), the collection (data loading and conversion), the analysis (description of the bibliometric analysis, creation of the attribute matrix), the visualization (Data reduction, Cluster, creation of a network matrix: bibliographic coupling, cocitation, collaboration, co-occurrence, and historiographical analysis), and the interpretation (mapping: figures, graphs, factorial maps, semantic maps, and network maps). In the present study, the five phases used by the latter authors were considered (Figure 1).

### 2.1 Literature search

Initially, we conducted a literature search in the Scopus database. This bibliographic database was created by the Publisher Elsevier in November 2004, currently has 42180 journals belonging to more than 7000 international publishers that summarizes the world's scientific production in various fields of knowledge (science, medicine, technology, arts, humanities, and social sciences). This database has several functions that facilitate the performance of bibliometric analysis.

The search strategy was performed through the title or title/abstract with some restrictions to reduce false positive results. The keywords were obtained from literature already published in psychological capital or Psycap. There were no restrictions on document type or language (although the title, abstract and keywords had to have an English language translation). The online data search and retrieval was performed on June 27, 2021. A search string was used with the Boolean operators “AND” and “OR”. The initial query without the application of any filter yielded 1390 documents, subsequently they were filtered, and the inclusion and exclusion criteria were applied. The combination of keywords, operators, and inclusion and exclusion criteria are presented in Table 1.

**Table 1:** Inclusion and exclusion criteria used for study identification.

	Criteria	Observation
Inclusion criteria	Documents that contain one of the keywords in the title, abstract, or keywords.	The present study contemplated the search for keywords concatenated with the following operators: “*OR”* or “*AND”*: “Psychological capital” OR “psycap” AND «University students» OR «college students»
	Documents written in any language whose title, abstract and keywords had an English translation.	In the present study, studies written in any language were considered, although those that had a translation of the title, abstract and keywords in the English language were considered.
	All dates	The search date was not specified, as the interest was focused on identifying the trend in terms of publications on psychological capital in the academic context of university students.
Exclusion Criteria	Documents addressing psychological capital in populations other than college students	This study contemplates the selection of documents that have addressed the psychological capital in the population of university students. Studies in other populations were excluded.
	Documents that address the academic psychological capital in university teachers or primary or secondary students.	Studies in primary and secondary school students and in teachers or administrative staff of educational institutions were excluded.

### 2.2 Data collection

It should be noted that this bibliometric review is oriented to psychological capital in university students as defined by the title of this work, therefore, studies that address academic psychological capital in university professors were not included. Based on the criteria presented in Table 1, a total of 1390 studies referring to psychological capital were collected. Then, these studies were refined with the words “psychological capital” OR “psycap” AND “university” AND “students” OR “college” AND “students” managing to identify 82 studies that met the inclusion and exclusion processes. These data were exported for analysis in BibTex format, since it is possible to import them into the Biblioshiny to be used in the Bibliometrix tool of the statistical program RStudio in its version R 4.1.1 (Aria and Cuccurullo, 2017).

### 2.3 Data analysis

For the analysis of the data, the Bibliometrix software was used for the statistical package RStudio. This is open *source software* created by Aria and Cuccurullo (2017) written in R language, which has a wide set of tools that allows bibliometric reviews through algorithm and scientific cartography useful in quantitative research. From version 2.0 a web interface called Biblioshiny has been incorporated, which allows the researcher without knowledge in programming to perform bibliometric analyses, in addition to performing data filters. Likewise, the VOSviewer 1.6.18 software was used to visualize the maps of keywords and cocitation of authors from the data obtained. Table 2 presents a synthesis of the information collected from the database.

**Table 2:** Synthesis of the information collected in Scopus.

Description of the data collected	Results
Time	2009:2021
Sources (journals, books, etc.)	56
Documents	82
Average years since publication	2,13
Average citations per document	4,56
Average citations per year for documents	1,09
References	3975
Keywords (ID)	174
Author's keywords (DE)	259
Types of documents	
Article	72
Book chapter	1
Conferences	7
Errata	1
Note	1
Authors	
Authors	206
Author's appearances	230
Single-author document authors	19
Authors of multi-author documents	187
Collaboration of authors	
Single-author documents	20
Documents by author	0,398
Authors by documents	2,51
Co-authors by documents	2,8
Collaboration Index	3,02

The data presented in Table 2 indicate that the Article type documents (*n* = 72) are the ones with the greatest presence in the database in the period between 2009 and 2021 according to the selected theme, followed by conferences (*n* = 7), book chapters, errata, and notes (*n* = 1, each type of document). The number of authors of the papers was 206, with an average citation per document of 4.56 and an author collaboration index of 3.02.

To perform the data analysis, the following categories of analysis were considered: type of document, annual scientific production, scientific journals, number of articles per author, ranking of author dominance, author keywords, citations of articles, production by country and institutions, collaboration map by country and the collaboration network by country. For the present study, the threshold of ten most productive authors, countries, and institutions, as well as the most cited journals and documents, was taken as a reference. This choice was made arbitrarily taking into account bibliometric studies already published (Farooq et al., 2021).

## 3 Results

The analysis of the data represents different aspects in the research, which includes the main countries and organizations with the highest production, as well as the main journals that produce publications on psychological capital in university students.

### 3.1 Annual scientific production (2009–2021), the 10 most influential countries and institutions

**Figure 2:**
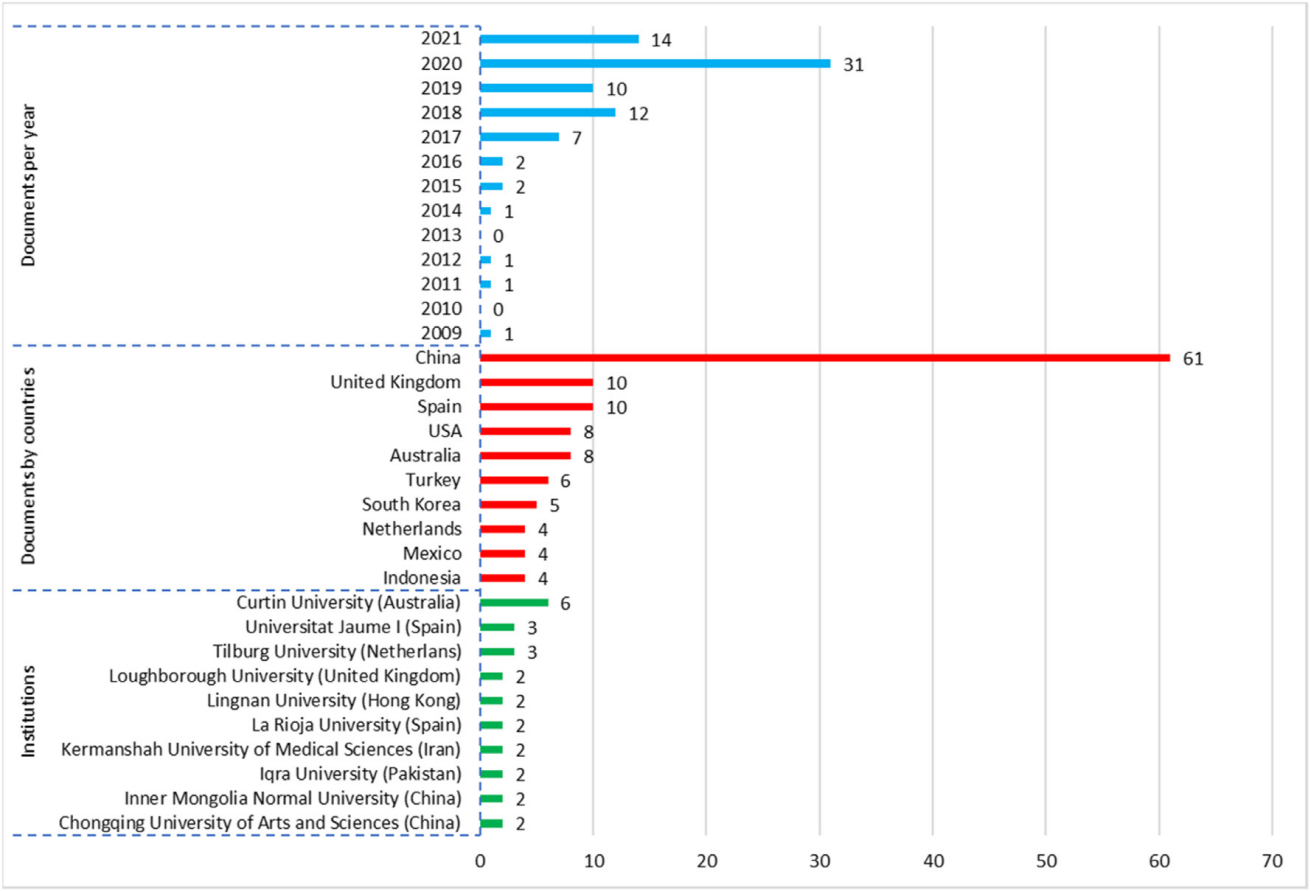
Annual scientific production (2009–2021), the 10 most influential countries and 10 institutions in the literature on psychological capital in university students.

Figure 2 shows the distribution of scientific production on psychological capital in university students worldwide between 2009 and 2021 (June), also shows the most influential countries and institutions in terms of the number of documents produced. In total, 82 studies were published, which denotes a low scientific production on the subject. 2020 was the year with the most documents published with a total of 31 investigations (Caballero et al., 2020; Cheung et al., 2020; Kornas-Biela et al., 2020; Liang et al., 2020; Prihatsanti et al., 2020), while between 2009 and 2016 the average of 1 document per year was published. However, there is a notable increase from 2017 (*n* = 7), followed by 2018 (*n* = 12), 2019 (*n* = 10), 2020 (*n* = 31) until June 2021 (*n* = 14), which shows an interest on the part of the scientific community in wanting to expand knowledge on this subject.

The most influential countries in terms of production were China with 61 documents (Bi and Jin, 2021; Cui, 2021; Kang et al., 2021; Shan et al., 2021; Xu and Zhang, 2021), followed by Spain, United Kingdom, Australia and United States with 10, 10, 8, 8 documents respectively. As for institutions of affiliation of the authors, the universities of Curtin, Tilburg, Jaume I and of Arts and Sciences of Chongqing stand out. Localized publications were written mainly in English (*n* = 78), followed by Chinese (*n* = 2), Spanish and Portuguese (*n* = 1, respectively).

### 3.2 The ten most influential journals

Table 3 presents the 10 most influential journals in the production of knowledge on the subject, including the “*Revista Argentina de Clínica Psicológica*” (N = 10), followed by *Current Psychology* (N = 5), *Frontiers in Psychology* (N = 5), *Boletín Técnico/Technical Bulletin* (N = 3) and *Journal of Happiness* Studies (N = 3). Of this group of journals, those that have received the highest citation (TC) were *Journal of Happiness Studies* (Quartile 1; TC = 30), *Education and Training* (Quartile 1; TC = 19), *Journal of Career Development* (Quartile 2; TC = 19), *Frontiers in Psychology* (Quartile 2; TC = 9) and *Current Psychology* (Quartile 2; CT = 8). Three of these magazines belonged to the United Kingdom, two to the United States, while Switzerland, Argentina, the Netherlands, China, and Venezuela had one magazine each. The two Latin American journals contained in the list were discontinued from the Scopus database and had no citation.

**Table 3:** The ten most influential journals in the literature on psychological capital in university students.

Ranking	Journal	TP	TC	H-Index	FI	Quartile	Publisher	Country
1	Revista Argentina de Clínica Psicológica	10	0	10	–	–	Fundación Aigle	Argentina
2	Current Psychology	5	8	41	0.498	Q2	Springer New York	USA
3	Frontiers in Psychology	5	9	110	0.947	Q2	Frontiers Media S. A.	Switzerland
4	Boletin Tecnico/Technical Bulletin	3	0	5	–	–	Universidad Central de Venezuela	Venezuela
5	Journal of Happiness Studies	3	30	73	1.198	Q1	Springer Netherlands	Netherlands
6	Academic Journal of Second Military Medical University	2	0	9	0.118	Q4	Second Military Medical University Press	China
7	Education and Training	2	19	65	0.743	Q1	Emerald Group Publishing Ltd.	United Kingdom
8	Journal of Career Development	2	19	45	0.518	Q2	SAGE Publications Inc.	USA
9	Journal of Small Business and Entrepreneurship	2	2	28	0.417	Q2	Taylor and Francis Ltd.	United Kingdom
10	Nurse Education Today	2	5	78	1.400	Q1	Churchill Livingstone	United Kingdom

***Note:*** TP: total publications; TC: total citations; FI: impact factor.

### 3.3 Most influential publications

In total, publications on psychological capital in university students received 374 citations in the Scopus database, 45 articles received at least one citation. The average citation per year of the top ten articles ranged from 3.0 to 10.25. Table 4 shows the list of the ten most influential publications, along with total citations, average citation per year, and document type.

**Table 4:** Most influential publications on psychological capital in university students.

Titles	Authors	Year	Journal	TC	TC (year)	TD
«Psychological Capital Among University Students: Relationships with Study Engagement and Intrinsic Motivation»	(Siu et al., 2014)	2014	“Journal of Happiness Studies”	82	10.25	Original article
«Enhancing Psychological Capital and Personal Growth Initiative: Working on Strengths or Deficiencies»	(Meyers et al., 2015)	2015	“Journal of Counseling Psychology”	37	5.28	Original article
«The relationship among college students' psychological capital, learning empowerment, and engagement»	(You, 2016)	2016	“Learning and Individual Differences”	30	5.00	Original article
«Ethnic Identity and Job Attribute Preferences: The Role of Collectivism and Psychological Capital»	(Combs et al., 2012)	2011	“Journal of Leadership & Organizational Studies”	23	2.30	Original article
«Psychological Capital and Performance among Undergraduate Students: The Role of Meaning-Focused Coping and Satisfaction”	(Ortega-Maldonado and Salanova, 2018)	2018	“Teaching in Higher Education”	22	5.50	Original article
«Negative Life Events and School Adjustment among Chinese Nursing Students: The Mediating Role of Psychological Capital»	(Liu et al., 2015)	2015	“Nurse Education Today”	21	3.00	Original article
«Predicting the Mental Health of College Students with Psychological Capital»	(Selvaraj and Bhat, 2018)	2018	“Journal of Mental Health”	20	5.00	Original article
«The Role of Psychological Capital in Academic Adjustment Among University Students»	(Hazan Liran and Miller, 2017)	2017	“Journal of Happiness Studies”	19	6.33	Original article
«Antecedents of Academic Performance of University Students: Academic Engagement and Psychological Capital Resources»	(Martínez et al., 2019)	2019	“Educational Psychology”	17	5.66	Original article
«Academic expectation, self-compassion, psychological capital, social support and student wellbeing»	(Poots and Cassidy, 2020)	2020	“International Journal of Educational Research”	9	4.50	Original article

### 3.4 List of 10 most productive authors

**Figure 3:**
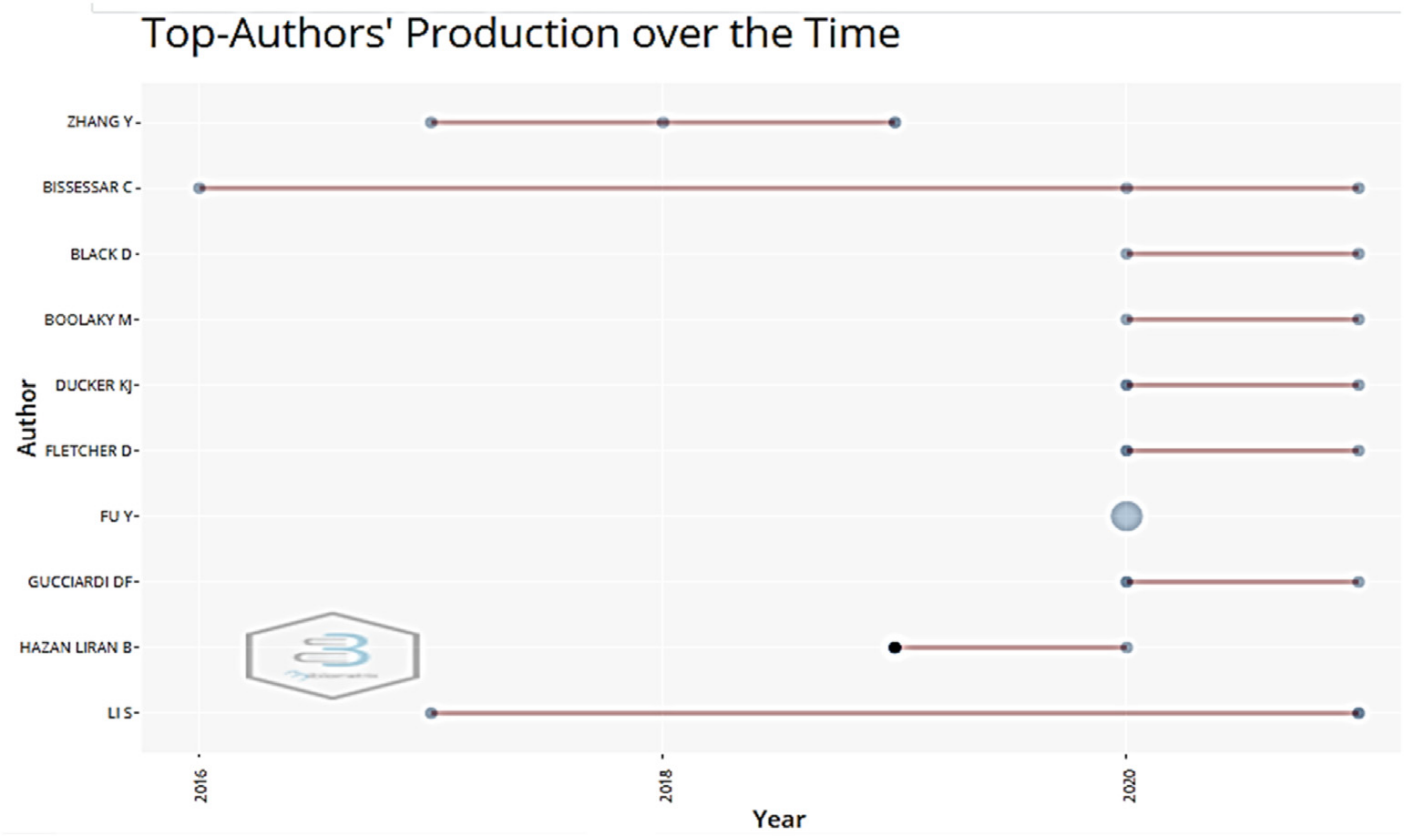
List of the ten authors with the most production over time.

Of the total of 82 selected documents, the top 10 authors concentrate 27% of the publications on the subject. The authorship patterns were as follows: one author (20 publications), three authors (20 publications), two authors (17 publications), four authors (15 publications), five authors (6 publications), six or more authors (4 publications). Table 5 shows the most productive authors, and Figure 3 reflects the production of these authors over time, highlighting a greater production between the years 2020–2021.

**Table 5:** List of 10 most productive authors.

Ranking	Authors	Documents	% 82	H-Index	Affiliation	TC
1	Zhang, Y.	3	3.65	1	Air Force Medical University	2
2	Bissessar, C.	3	3.65	-	University of Guyana	0
3	Black, D.	2	2.43	1	University of Liverpool Online	0
4	Boolaky, M.	2	2.43	-	University of Lincoln	0
5	Ducker, K. J.	2	2.43	1	Curtin University	2
6	Fletcher, D.	2	2.43	1	Loughborough University	2
7	Fu, Y.	2	2.43	-	Yangtze University	0
8	Gucciardi, D. F.	2	2.43	1	Curtin University	2
9	Hazan Liran, B.	2	2.43	1	University of Haifa	19
10	Li, S.	2	2.43	1	Renmin University of China	2

### 3.5 Co-occurrence network of author keywords

**Figure 4:**
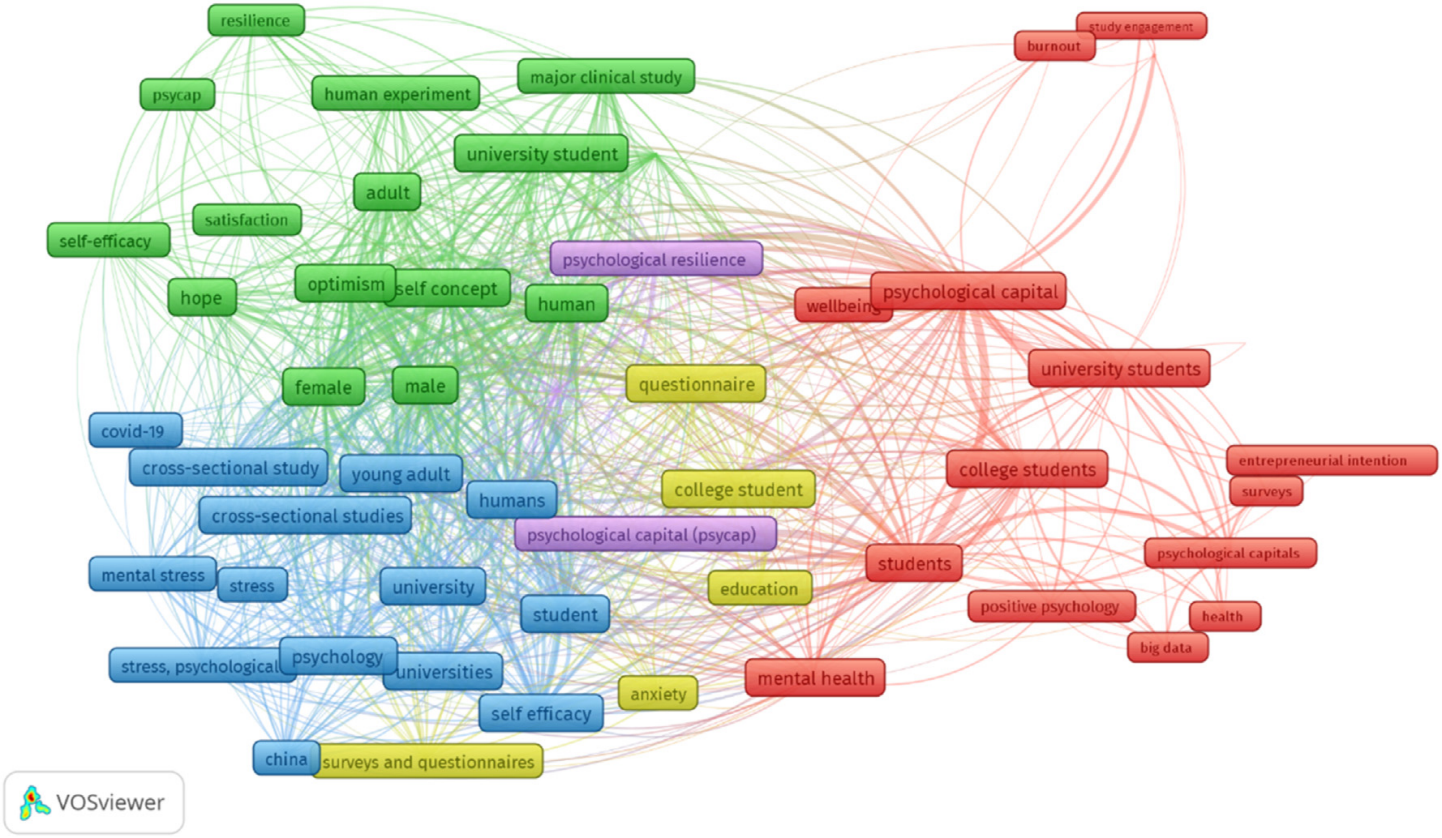
Co-occurrence network of author keywords.

Figure 4 shows the author's keyword co-occurrence network, which is organized into five interlocking clusters of keywords, whose themes were:

*Cluster 1 (blue),* with 17 keywords related to the following themes: “stress”, “covid-19”, “young adults”, “self-efficacy”, “students” and “university”; in this cluster, cross-sectional and survey studies related to capital and psychological variables associated with the current pandemic in young university adults were carried out.

*Cluster 2 (red),* with 19 keywords such as “academic performance”, “burnout”, “well-being”, “entrepreneurship intention”, “academic commitment”, “well-being” and “mental health”; in this group, studies of survey and *big data* were carried out that relate psychological capital with academic and health results in university students.

*Cluster 3 (green),* with 16 keywords such as “hope”, “optimism”, “resilience”, “self-concept”, “self-efficacy”, “satisfaction” and “self-concept”; in this grouping controlled and experimental clinical studies were carried out considering the dimensions of psychological capital as well as associated educational and psychological variables.

*Cluster 4 (yellow),* with five keywords such as “anxiety”, “education”, “university students”, “questionnaires”, and “surveys” (Al-Dwaikat et al., 2020; Castaños-Cervantes and Domínguez-González, 2020).

*Cluster 5 (purple),* with three keywords such as “psychological capital (*psycap*)”, “psychological resilience” and “resilience”; in this cluster, studies were applied that explore psychological resilience as a dimension of psychological capital in university students.

### 3.6 Trend topics

**Figure 5:**
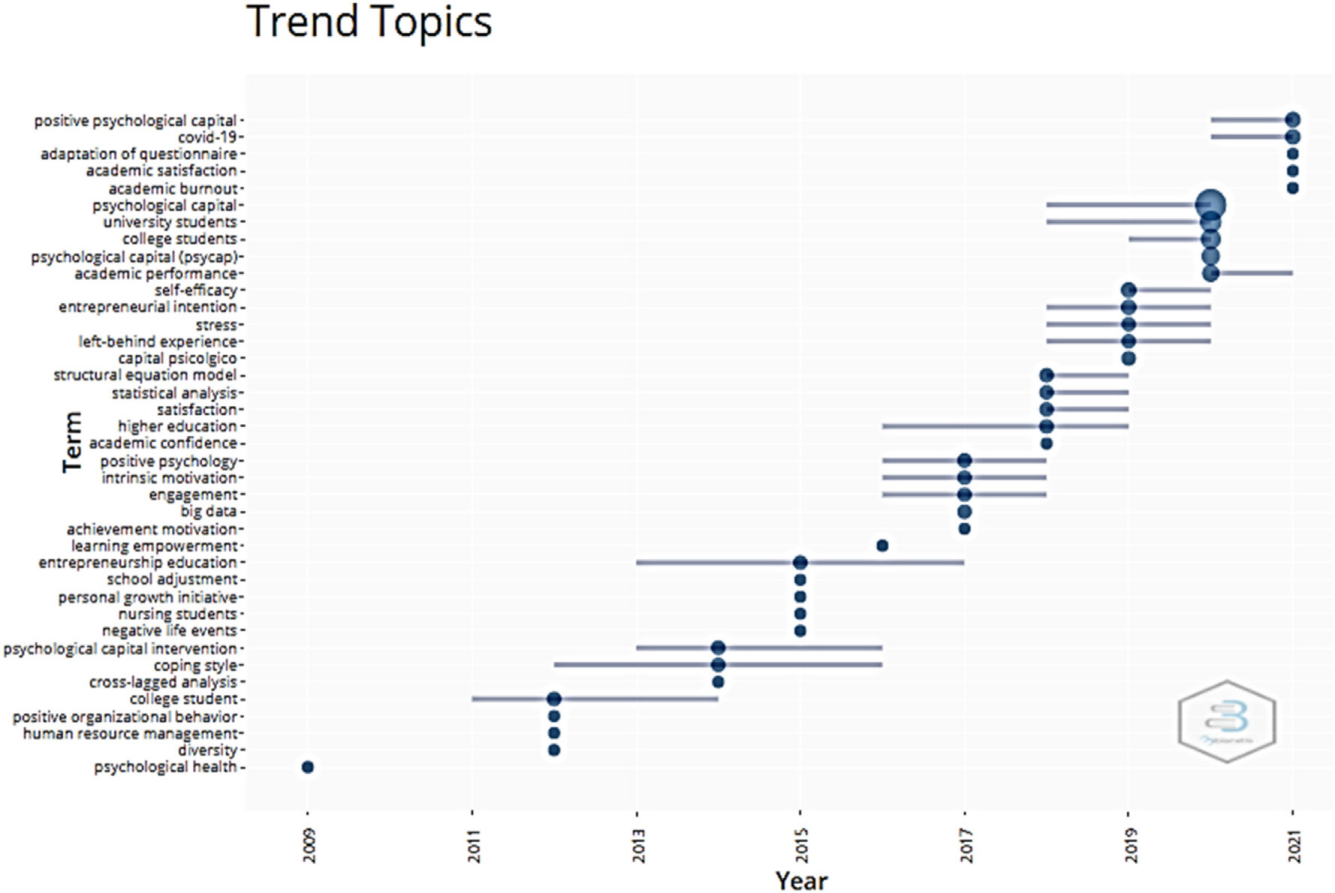
Trend topics around psychological capital in university students (2009–2021).

Figure 5 shows the trend topics in the study of psychological capital in university students from 2009 to 2021. There is evidence of an increase in research in the last five years that has explored the relationship between psychological capital and variables such as academic satisfaction, academic performance, entrepreneurship intention, academic confidence, motivation, and academic commitment. For the year 2021, studies in the context of the covid-19 pandemic, academic satisfaction, academic exhaustion and adaptation of questionnaires stand out (Aryani et al., 2021; Karing, 2021; Ma and Liu, 2021; Villanueva-Flores et al., 2021; Wang et al., 2021).

### 3.7 Analyses that relate the author's keywords, countries, and institutions

**Figure 6:**
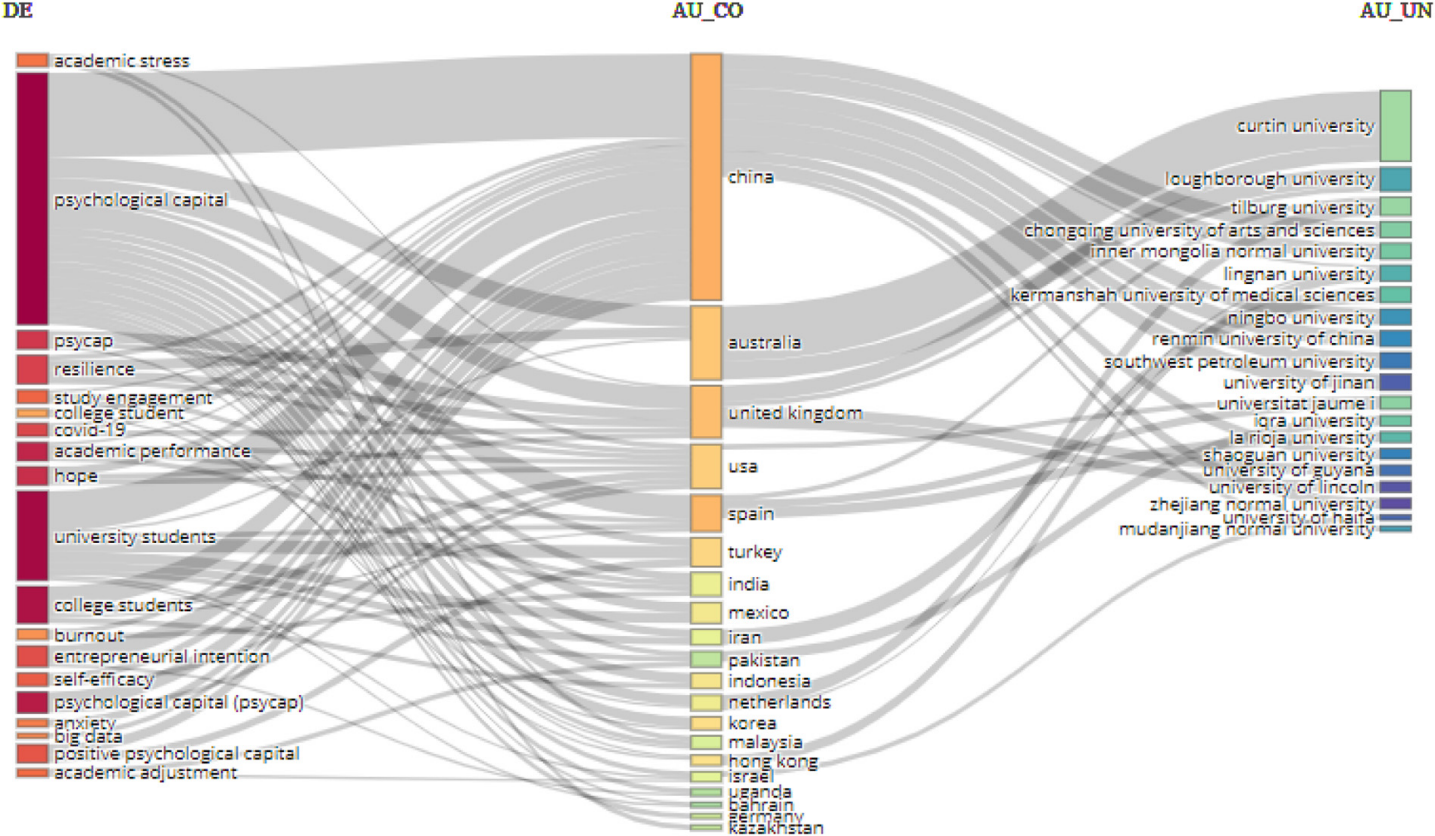
Analyses that relate the author's keywords, countries, and institutions.

Figure 6 presents the analysis of three factors, which relate keywords (left), countries (center) and institutions of affiliation of the authors (right). They are studies closely related to the author's keywords (academic stress, psychological capital, *psycap*, academic commitment, resilience, and university students) and that were developed by countries such as China, Australia, United Kingdom, Spain, and Iran). These keywords and countries have a close link with three of the authors' main affiliate institutions (Curtin University, Loughborough University, Tilburg University).

### 3.8 Map of collaboration between countries

**Figure 7:**
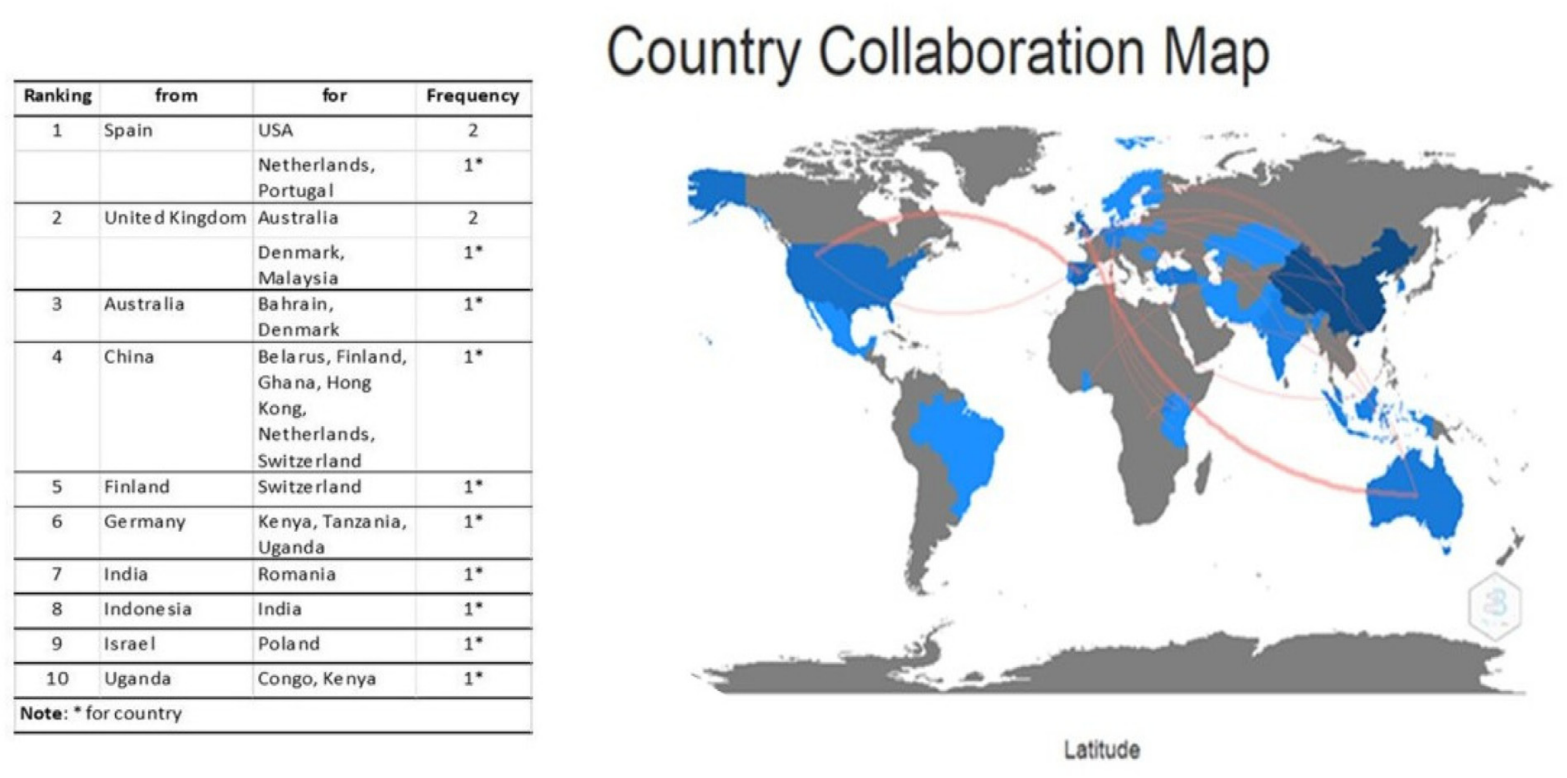
Map of collaboration between countries.

The analysis of collaboration in international research showed that, of the 82 publications, only 20 (24%) were made with international collaboration. Of the ten most active countries, China showed the highest percentage of documents with international collaboration, followed by Spain, Australia, the United Kingdom, Germany, and Uganda (Figure 7).

### 3.9 Authors' citation network

**Figure 8:**
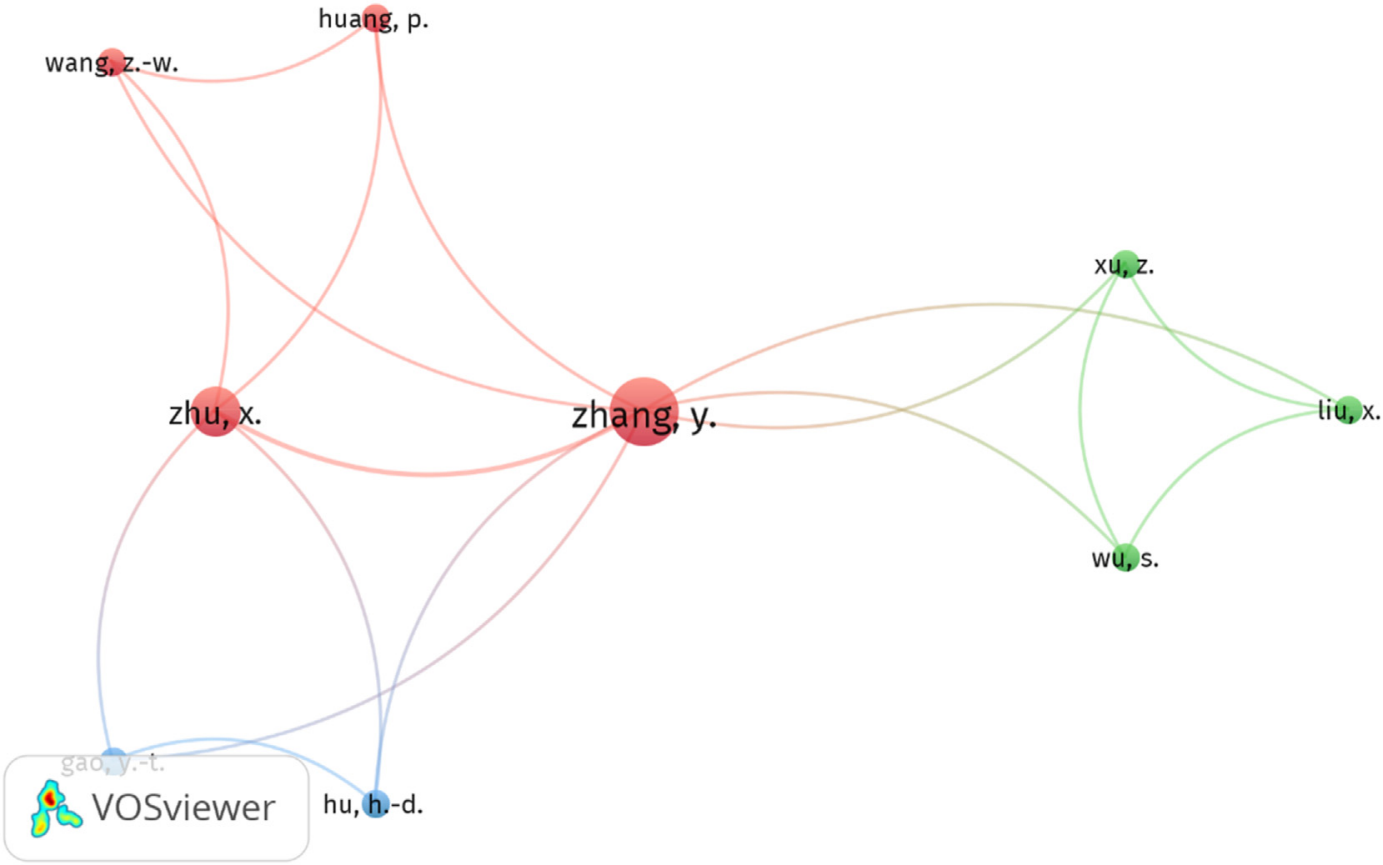
Authors' citation network.

The map of authors' citation networks with a minimum research result of 2 papers showed three groups of cocitation (Figure 8). The first group (red color) included four authors led by Zhang Y. and Zhu X., and is linked to the second group (blue), led by authors Gao Y. and Hu H., and the third group (green) is led by authors Xu Z., Liu X and Wu S.

## 4 Discussion

Psychological capital (PsyCap) is in a unique position at the intersection between the student and the academic institution. It plays a crucial role in improving academic outcomes and is an important predictor of student performance and educational organizations, although unfortunately little has been studied in these contexts (Adil et al., 2020). The present study aimed to analyze the main bibliometric indicators of scientific production, collaboration and impact of the scientific documents contained in the Scopus database, related to psychological capital in university students.

This research is the first Review (to date of completion) that allows the mapping of available information on psychological capital in university students. As for the volume of scientific production of 82 documents, this has its lowest level between the years 2009 and 2019 except for the years 2020 and 2021. China leads the scientific production in the subject with 61 documents, 5 times above the second and third place that is occupied by Spain and the United Kingdom with 10 publications each. The institutions of affiliation of the authors that stand out in the greatest number of productions are the Universities of Curtin with 6 documents and Tilburg and Jaume I with 3 documents respectively.

The most influential journals in terms of production were the *Revista Argentina de Clínica Psicológica* (N = 10), followed by *Current Psychology* (N = 5) and *Frontier in Psychology* (N = 5). Although these journals have produced a greater number of publications, the impact they have had in terms of citation is low or none in some cases, compared to journals such as *the Journal of Hapiness Studies*, *Education and Training* and *Journal of Career Development*, which have had a low production, but have received a greater number of citations. Of the total of the most productive journals, two of them, located in Latin American countries, have been disincorporated from the Scopus database and have not received a citation until the time of the review. This imposes a responsibility on Latin American institutions and researchers to finance and carry out significant and impactful research worldwide in terms of the subject, considering that each time the scientific production around psychological capital continues to increase in recent years; It is also considered a psychological resource that can increase academic, health and well-being outcomes in university students.

Regarding the pattern of authorship, publications with single authors and three authors stand out, followed closely by two authors, four authors and five authors respectively, with a ratio of 2.51 authors per document and 0.398 documents per author. The most productive authors were Zhang Y. and Bissessar C. with three publications each. The ten most productive authors concentrated 27% of the publications on the subject. On the other hand, the analysis of collaboration between authors highlights a collaboration index of 3.02 and 2.8 co-authors per document. Of the 10 most active countries, China showed the highest percentage of documents with international collaboration, followed by Spain, the United Kingdom and Australia. The authors' citation network analysis resulted in three groups of authors, where Zhang Y. and Zhu X. show the highest cocitation.

The most influential publication in relation to psychological capital in university students was made by Siu et al. (2014), in the *Journal of Happiness Studies*, which has had a total of 82 citations and an average citation per year of 10.25. The most influential topics in the first 10 journals relate psychological capital to commitment to studies, intrinsic motivation, personal growth, empowerment in learning, ethnic identity, work attributes, collectivism, academic performance, academic satisfaction, coping, negative life events, academic adjustment, mental health, academic expectations, self-compassion, social support, and well-being, which can be guiding topics for academics dedicated to the study of this subject.

For the co-occurrence network analysis, the topics of trends and keyword analysis of the author highlight 5 clusters with topics related to anxiety, covid-19, stress, self-efficacy, academic performance and exhaustion, academic commitment, entrepreneurship intention, mental health and well-being, satisfaction, self-concept, psychological resilience, among others (Piepiora and Szczepańska, 2020). Localized studies have sometimes linked psychological capital with constructs in the academic area, psychological and occupational health, the latter in pre-professional exhibitions and university students. This is due to the narrowly defined difference between young adults and the labour force; however, university students live in a quasi-professional and academic environment where they have expectations about the academic and work future, in addition to expecting to achieve objectives, goals and have tasks to perform where their performance is evaluated and rewarded in a regular, tangible, and intangible way. The information search for this bibliometric research was limited to a single database called Scopus.

## 5 Conclusions

To conclude, 2020 was the year with the highest production of documents on the subject under study with 31 investigations. China is the country with the highest number of documents on Psychological Capital in university students with 61 articles. The institution with the most manuscripts on the subject is Curtin University with 6 documents. The most influential journal is the “Revista Argentina de Clínica Psicológica” with 10 documents; and the authors of greatest production were Zhang, Y. and Bissessar, C. with 3 articles for each. By June 2021, the date of this bibliometric review, this document would be the first of the “Review” type according to the data obtained from the Scopus database (see Table 2).

It is suggested to develop future research with topics such as: (a) psychological capital and coping style, (b) psychological capital and entrepreneurial spirit, (c) psychological capital and academic commitment and (d) positive psychological capital characterized by self-efficacy, optimism, perseverance, and resilience.

Thus, this study can help both academics and professionals to obtain an overview of the advances in the subject and thus identify important gaps in the literature and propose promising new lines of research.

## Declarations

### Author contribution statement

All authors listed have significantly contributed to the development and the writing of this article.

### Funding statement

This research did not receive any specific grant from funding agencies in the public, commercial, or not-for-profit sectors.

### Data availability statement

Data included in article/supp. material/referenced in article.

### Declaration of interest's statement

The authors declare no conflict of interest.

### Additional information

No additional information is available for this paper.
